# Autologous hematopoietic stem cell transplantation for multiple myeloma in the age of CAR T cell therapy

**DOI:** 10.3389/fonc.2024.1373548

**Published:** 2024-03-27

**Authors:** Charlotte F. M. Hughes, Gunjan L. Shah, Barry A. Paul

**Affiliations:** ^1^ Adult Bone Marrow Transplant Service, Memorial Sloan Kettering Cancer Center, New York, NY, United States; ^2^ Cellular Therapy Service, Memorial Sloan Kettering Cancer Center, New York, NY, United States; ^3^ Department of Hematologic Oncology and Blood Disorders, Levine Cancer Institute, Atrium Health/Wake Forest Baptist, Charlotte, NC, United States

**Keywords:** autologous transplant, CAR T cell therapy, multiple myeloma, newly diagnosed multiple myeloma, relapsed refractory multiple myeloma

## Abstract

Chimeric antigen receptor (CAR) T cell therapy has revolutionized the management of relapsed and refractory myeloma, with excellent outcomes and a tolerable safety profile. High dose chemotherapy with autologous hematopoietic stem cell transplantation (AHCT) is established as a mainstream of newly diagnosed multiple myeloma (NDMM) management in patients who are young and fit enough to tolerate such intensity. This standard was developed based on randomized trials comparing AHCT to chemotherapy in the era prior to novel agents. More recently, larger studies have primarily shown a progression free survival (PFS) benefit of upfront AHCT, rather than overall survival (OS) benefit. There is debate about the significance of this lack of OS, acknowledging the potential confounders of the chronic nature of the disease, study design and competing harms and benefits of exposure to AHCT. Indeed upfront AHCT may not be as uniquely beneficial as we once thought, and is not without risk. New quadruple-agent regimens are highly active and effective in achieving a deep response as quantified by measurable residual disease (MRD). The high dose chemotherapy administered with AHCT imposes a burden of short and long-term adverse effects, which may alter the disease course and patient’s ability to tolerate future therapies. Some high-risk subgroups may have a more valuable benefit from AHCT, though still ultimately suffer poor outcomes. When compared to the outcomes of CAR T cell therapy, the question of whether AHCT can or indeed should be deferred has become an important topic in the field. Deferring AHCT may be a personalized decision in patients who achieve MRD negativity, which is now well established as a key prognostic factor for PFS and OS. Reserving or re-administering AHCT at relapse is feasible in many cases and holds the promise of resetting the T cell compartment and opening up options for immune reengagement. It is likely that personalized MRD-guided decision making will shape how we sequence in the future, though more studies are required to delineate when this is safe and appropriate.

## 1 Introduction

Multiple myeloma (MM) is a cancer of terminally differentiated plasma cells in the bone marrow. For 2023, an estimated 35,730 new cases will be diagnosed in the United States which represents 1.8% of all cancers and 19% of all hematologic malignancies (1). The advent of high doses of melphalan with autologous hematopoietic stem cell transplantation (AHCT) was a major advance and led to improved response rates (RR), progression free survival (PFS), and, in some trials, prolonged overall survival (OS) in patients with newly diagnosed MM (NDMM) and has been a cornerstone of treatment in eligible patients for the last 20 years (2–6). While there are no universally agreed upon transplant eligibility criteria and several risk stratification tools have been proposed, factors such as age, baseline performance status, and comorbidities are important tenets in determining a patient’s eligibility (7–10). Recently, adoptive cell therapy using BCMA-directed autologous chimeric antigen receptor (CAR) T therapies have been tested in patients with relapsed refractory multiple myeloma (RRMM) and have shown unprecedented response rates, depth of response, and improved PFS when compared to standard of care (SOC) regimens (11–14). Currently, CAR T cells are under investigation for use as consolidation after induction therapy in transplant ineligible patients and are being compared head-to-head against high-dose melphalan and AHCT in large phase III trials of transplant eligible patients (15, 16). While these trials are yet to report, there is significant reason to believe that CAR T therapy may lead to improved outcomes as T-cell fitness - which is a prime driver of CAR T success - has been shown to decline with increasing lines of MM directed treatment (17–20). This also leads to the question of whether these therapies should be sequenced or are mutually exclusive. In this manuscript, we review the current data for each of these modalities and discuss trials currently evaluating AHCT and CAR Ts for patients with MM. Finally, we provide our thoughts on the role of each of these treatments in MM therapy in the United States where both options are commercially available.

## 2 CAR T in MM

CAR T cell therapies have revolutionized the treatment of RRMM. Most CAR T target B cell maturation antigen (BCMA) also known as TNFRSF17 or CD269; a type III transmembrane glycoprotein and non-tyrosine kinase receptor in the tumor necrosis factor receptor (TNFR) superfamily (21, 22). Expression of BCMA is selectively induced during plasma cell differentiation. Expression is nearly absent on naïve and memory B cells but is ubiquitously expressed on plasmablasts and plasma cells (23–25). BCMA expression is rare in other tissues with only low-level BCMA mRNA and protein expression seen in areas with endogenous plasma cell populations (i.e., the testes, gastrointestinal tract and trachea) (25). Additionally, expression of plasma cell BCMA increases with progression from monoclonal gammopathy of undermaintained significance (MGUS), to smoldering multiple myeloma (SMM) and MM. Higher levels of soluble BCMA has been associated with shorter time to progression in MGUS and SMM patients, and higher levels surface BCMA is associated with worse prognosis in MM patients (26–31). Several different modalities (antibody drug conjugates, bispecific antibodies, and CAR T) have been designed to target BCMA. Two BCMA CAR T: idecabtagene vicleucel (ide-cel) and ciltacabtagene autoleucel (cilta-cel) have been FDA approved for RRMM based on outcomes seen in large phase 2 trials.

### 2.1 Idecabtagene vicleucel

Ide-cel is a second-generation CAR which uses a lentiviral vector to transduce a BCMA targeting scFv fused to a 4-1BB co-stimulatory and CD3 signaling domains (32, 33). The pivotal phase II KarMMa study evaluated ide-cel at various doses in 128 RRMM patients who had previously received ≥ 3 prior lines of therapy including an immunomodulatory drug (IMiD), proteasome inhibitor (PI), and an anti-CD38 monoclonal antibody. Patients were infused with 150×10^6^ to 450×10^6^ CAR T cells. Overall response rate (ORR) was 73%, with 42 (33%) patients achieving a complete response (CR) or better. Measurable residual disease (MRD) negativity (at 10^-5^) was achieved in 26% of all patients, but 79% of patients who achieved a ≥ CR. Notably, these response rates were relatively preserved across patients with high-risk features including penta-refractory disease, extramedullary disease, and high-risk cytogenetics. Median PFS was 8.8 months, but was 20.2 months in patients who achieved ≥ CR (11). OS for the KarMMa study was 24.8 months, but interestingly, was lower in the cohort of patients who had previously been treated with 3 prior lines (median OS 22 months) versus those who had been treated with 4 or more prior lines (median OS 25.2 months) (34). Based on these findings, ide-cel was approved for the treatment of adults with RRMM after four or more prior lines of therapy including an IMiD, PI, and anti-CD38 monoclonal antibody by the US Food and Drug Administration (FDA) in March 2021. In a *post-hoc* analysis of the KarMMa trial, lower levels of serum soluble BCMA, more robust blood and bone marrow CAR T expansion, and an increased ratio of naive and early memory CD4 T cells compared to senescent CD3 and CD8 T cells in the apheresis product prior to manufacturing were associated with improved response to ide-cel (35). Subsequently, real-world data for patients treated with commercial ide-cel outside the context of a clinical trial showed very similar efficacy. ORR in this population was 84% with 42% achieving ≥ CR. Median PFS (8.5 months) at 6.1 months of follow-up was similar to the KarMMa data. Notably, 75% of the patients included in this cohort would have been deemed ineligible to enroll on the KarMMa trial (36). Predictors of poor response to ide-cel in the real-world cohort included prior BCMA therapy, high-risk cytogenetics, elevated baseline ferritin level, and younger age (36, 37).

More recently ide-cel was evaluated against investigator’s choice of one of 5 SOC regimens: daratumumab, pomalidomide, and dexamethasone; daratumumab, bortezomib, and dexamethasone; ixazomib, lenalidomide, and dexamethasone; carfilzomib and dexamethasone; or elotuzumab, pomalidomide, and dexamethasone in RRMM patients who had received 2-4 prior lines of therapy including an IMiD, PI, and an anti-CD38 monoclonal antibody in the phase III KarMMa-3 trial. This trial enrolled a substantial population (43%) of patients with high-risk cytogenetics (defined as presence of del17p, t[4;14], or t[14;16]) which were evenly distributed across both arms. The ORR was 71% for ide-cel and 42% for SOC. Median PFS in the intention-to-treat population was substantially higher in the ide-cel arm (13.3 months vs 4.4 months). The hazard ratio for disease progression or death for ide-cel vs SOC was 0.49 (95% CI 0.38 - 0.65). Ide-cel showed similar improved hazard ratios for disease progression or death in patients with high-risk cytogenetics (HR 0.61; 95% CI 0.41-0.90), extramedullary disease (HR 0.40; 95% CI 0.25-0.65), and disease refractory to at least one IMiD, PI, and anti-CD38 monoclonal antibody (HR 0.46; 95% CI 0.34-0.62) (14, 38). The FDA is currently reviewing a supplemental biologics license for the approval of ide-cel in this less heavily pretreated population based on the data from the KarMMa-3 trial. Additionally, cohort 2c of the phase II KarMMa-2 trial is evaluating the efficacy of ide-cel in patients with newly diagnosed MM (NDMM) who had an inadequate response to frontline AHCT. In this population, 77% of patients achieved ≥ CR, and median PFS has not be reached at a median follow-up of 39.4 months (39).

### 2.2 Ciltacabtagene autoleucel

Similar to ide-cel, cilta-cel uses a lentiviral vector to create a construct with a CD3ζ activation domain, and 4-1BB costimulatory domain. Cilta-cel’s antigen binding domain contains bispecifc scFvs targeting two distinct BCMA epitopes, VHH1 and VHH2 (40). This bi-epitope binding confers higher avidity and specificity to BCMA. Cilta-cel was evaluated in the phase Ib/II CARTITUDE-1 trial RRMM patients who had previously been treated with ≥ 3 or were double refractory to an IMiD and a PI, and had previously received an anti-CD38 antibody. Ninety-seven patients were treated with cilta-cel (29 in the phase Ib portion, 68 in the phase II portion). The population was heavily pretreated (median of 6 prior lines; 84% penta-exposed) and included 42% who were penta-refractory (12). ORR was 98%, with 95% of patients achieving a VGPR or better and 82.5% of patients achieving a sCR. MRD negativity was evaluated in 61 patients at 10^-5^ and 52 patients at 10^-6^. MRD negativity rates were 92% and 75% at 10^-5^ and 10^-6^ respectively (41). Median PFS was 34.9 months; median OS has not been reached at 27.7 months of follow-up (42). Based on these data cilta-cel was approved for the treatment RRMM patients following 4 or more prior lines of therapy, including an IMiD, PI, and an anti-CD38 monoclonal antibody in Feb 2022. Real-world data for cilta-cel is not as mature as that for ide-cel. However, in an early analysis a multi-institutional cohort of 143 patients infused with commercial cilta-cel-of whom 57% would have been ineligible for participation in the CARTITUDE-1 trial-ORR was 84% with 53% ≥ CR. Notably, 22% of patients included in this dataset were infused with an out of specification (OOS) cilta-cel product. The presence of high-risk cytogenetics (defined as the presence of del17p, t[4;14)], or t[14;16]) was associated with poorer ORR, PFS, and OS in multivariate analysis (43).

Cilta-cel was also evaluated in less heavily pretreated patients in the phase III CARTITUDE-4 trial. This trial randomized 419 RRMM patients previously treated with 1-3 prior lines to either cilta-cel or investigator’s choice of 2 SOC regimens: pomalidomide, bortezomib, dexamethasone, or daratumumab, pomalidomide, dexamethasone. The trial strongly favored the cilta-cel arm with ORR of 84.6% vs 67.3% for SOC. Responses were deeper in the cilta-cel arm (73.1% vs 21.8% ≥ CR) which translated into improved PFS (median PFS not reached for cilta-cel, 11.8 months for SOC; HR 0.26; 95% CI, 0.18 -0.38). Subgroup analysis also favored cilta-cel for all parameters tested including high-risk cytogenetics, prior lines of therapy, degree of refractoriness, and presence of extramedullary disease (13, 44). Cilta-cel is currently under evaluation for patient with suboptimal response to frontline transplant, in treatment naïve high-risk and standard risk NDMM in cohorts D, E, F of the mutli-cohort CARTITUDE-2 trial.

### 2.3 CAR T toxicities

While these data illustrate the efficacy of both ide-cel and cilta-cel, both of these agents have important toxicities that need to be factored into any suitability discussion. Cytokine release syndrome (CRS) is a systemic inflammatory response thought to be secondary to activation of bystander immune and nonimmune cells resulting in significant cytokine release-especially IL-1, IL-2, IL-6, GM-CSF, and IFN-g (45–48). This inflammatory storm typically manifests as fever, fatigue, headache, arthralgias, and myalgias, but higher-grade manifestations including hypotension, shock, disseminated intravascular coagulation, and multiorgan system failure can occur (49, 50). Treatment of CRS ranges from supportive care with antipyretics, intravenous fluids, and supplemental oxygen for lower grade symptoms to vasopressors, the anti-IL-6 antibody tocilizumab, and high-dose corticosteroids for higher grade symptoms (50, 51). While low grade CRS is very common with both ide-cel and cilta-cel the timing of CRS onset varies for each CAR-T product (see **Table 1**). Ide-cel was associated with 84% CRS in the KarMMa trial with the majority being low grade (5% grade 3 or 4). Median onset of CRS with ide-cel in the KarMMa trial, the KarMMa-3 trial, and the real-world dataset was reliably 1 day (range 0-14) (11, 14, 36). In the CARTITUDE-1 trial 95% of patients experienced CRS with 4% grade 3 or 4. Median onset of CRS with cilta-cel in the CARTITUDE-1 trial was 7 days, while it was 8 days in the CARTITUDE-4 trial, and 9 days in the real-world cohort (12, 13, 43). These data are summarized in **Table 1**.

**Table 1:** Selected Toxicities in BCMA CAR-Ts.

Toxicity	Ide-cel (KarMMa)	Ide-cel (KarMMa-3)	Ide-cel (Real World)	Cilta-Cel (CARTITUDE-1)	Cilta-Cel (CARTITUDE-4)	Cilta-Cel (Real World)
**Number of prior Lines**	≥3	2-4	≥4	≥3	1-3	≥4
**ORR (%)**	73	71	84	97	85	84
**Median PFS (months)**	8.8	13.3	8.5	34.9	NR	NR
**% CRS (all; grade 3/4)**	84, 5	88, 4	82, 3	95, 4	76, 1	80, 5
**Median Onset of CRS (range)**	1 day (1-12)	1 day (1-14)	1 day (0-14)	7 days (5-8)	8 days (1-23)	9 days (1-23)
**% Neurotoxicity (all; grade 3/4)**	18, 3	15, 3	18, 6	17, 2	21, 3	18, 6
**Median Onset of ICANS (range)**	2 days (1-10)	3 days (1-317)	3 days (0-15)	8 days (6-8)	9.5 days (1-6)	9.5 days (1-6)
**% Delayed Neurotoxicity (all; grade 3/4)**	0	0	1	12, 8	NR	12, NR

ORR, Overall response rate; CRS, Cytokine release syndrome; ICANS, Immune effector cell associated neurotoxicity syndrome; NR, Not reached.

Immune effector cell-associated neurotoxicity syndrome (ICANS) is another adverse effect common to CAR-T therapy. ICANS is a toxic encephalopathy thought to be related to endothelial cell activation and disruption of the blood brain barrier mediated by inflammatory cytokines and chemokines, which results in direct neuronal cell injury. Mild symptoms include headache, confusion, focal neurologic deficits, and impaired fine motor skills. Higher grade ICANS can manifest with aphasia, seizure, cerebral edema, and coma (49). The mainstay of ICANS management is high-dose corticosteroids; additional supportive measures including mechanical ventilation (if evidence of airway compromise) may be needed. Tocilizumab is generally only used if patients have coexisting CRS (50, 51). ICANS typically manifests later than CRS (around day 2-3 with ide-cel and days 8-10 with cilta-cel) (11–14, 36, 43). An additional neurologic toxicity seemingly unique to cilta-cel is less well understood. Termed movement and neurocognitive treatment-emergent adverse events (MNTs), they compromise a cluster of movement (e.g., micrographia, tremors), cognitive (e.g., memory loss, disturbance in attention), and personality changes (e.g. reduced facial expression, flat affect) which typically manifest after symptoms of CRS and ICANS have resolved, and unlike ICANS, are generally not responsive to steroids (52, 53). While the unique AEs associated with CAR-Ts are certainly cause for concern, with increasing ubiquity of this class of therapy and earlier recognition of, and intervention for, CRS and ICANS the rates of higher-grade AEs are decreasing in more recent trials (summarized in **Table 1**). Ideally, less high-grade CRS and ICANS will lead to less delayed neurotoxicity and MNT events.

Finally, the risk of secondary primary malignancies, long known to be an adverse effect of several myeloma therapies is largely unknown post-CAR T. However, recent data does raise concern for a possible increased risk. Specifically, rare reports of T-cell lymphomas derived from CAR T cells have been reported in several CAR T recipients. To date, only 12 cases have been reported out of the 7946 patients infused with CAR Ts, indicated predominately for B-cell lymphomas (54). Only 1 case has been reported in a myeloma patient who was treated with cilta-cel, but analysis of the patient’s apheresis product (prior to CAR T manufacture) suggested that they had several genetic mutations present at baseline and the role of the CAR is unclear (55). Similarly, myeloid malignancies have been reported with post CAR T. In the long-term follow-up of the CARTITUDE-1 trial 9 patients (9%) have been diagnosed with MDS or AML (42). However, it is unclear whether this a result of the CAR T or lymphodepleting chemotherapy. To that effect, a recent analysis of 4 patients who developed MDS after treatment with an investigational anti-BCMA CAR T showed that while none of the patients had morphologic changes consistent with MDS prior to CAR T infusion, all four patients exhibited molecular alterations associated with MDS in their pre-CAR T as well as post-CAR T therapy bone marrow with no new mutations observed after CAR T (56). Clearly further follow-up is warranted.

## 3 Autologous hematopoietic stem cell transplant

High-dose chemotherapy with melphalan followed by AHCT is well established as standard of care for patients with NDMM who are sufficiently fit to tolerate intensive therapy, ie, are transplant eligible. The principle of this practice is to induce disease control and collect a clean stem cell product following a limited induction, then administer powerful anti-myeloma therapy which would only be feasible with a stem cell rescue. It was theorized and then confirmed that this would provide a PFS benefit if offered early in the treatment course, as opposed to deferring it until a later relapse. Foundational studies showed improved PFS and OS and were a gamechanger in the natural history of myeloma (2, 57, 58). Short-term treatment related adverse events include obligate cytopenias, as well as infections, gastrointestinal upset, and mucositis (5, 59, 60). Despite these acute effects, treatment-related mortality remains low, and though quality of life is impacted in the short-term, these effects appear to be transient and recover post AHCT (59, 61, 62).

When PI-IMiD combination therapy became standard care (63), new studies were required to update our understanding of the true benefit of upfront AHCT with modern regimens and are summarized in **Table 2**. The IFM 2009 trial published in 2017 (5), included induction with three cycles of lenalidomine, bortezomib, dexamethasone (RVd) and 1 year of maintenance lenalidomide after consolidation and demonstrated a PFS benefit, which was sustained in the updated long-term follow up data (64). No difference in OS was noted at 4 years. The DETERMINATION trial included maintenance until disease progression and demonstrated a greater PFS than its IFM precursor, and confirmed the PFS benefit of AHCT when added to RVd, but again did not show an OS advantage at 72 months follow-up (59). The FORTE study looked at new generation PI carfilzomib(K)-based regimens as induction and confirmed the PFS benefit of upfront AHCT with both KRd and KCd induction (65). In the CARDAMON trial published in 2022, investigators determined that PFS of KCd alone was not non-inferior to upfront AHCT following KCd induction at 2 years (66).

**Table 2:** Recent trials comparing upfront versus delayed AHCT in patients with NDMM.

	Induction regimen in AHCT group	PFS (months) (Upfront AHCT vs control)
**IFM-2009**	VRd	Median: 50 vs 36
**EMN-02/H095**	VCd	Median: 56.7 vs 41.9
**FORTE**	KRd*	3-year PFS: 56% vs 33%
**DETERMINATION**	VRd	Median: 67.5 vs 46.2
**Cardamon**	KCd	Median: 42.4 vs 33.8

VRd, bortezomib, lenalidomide, dexamethasone; VCd, bortezomib, cyclophosphamide, dexamethasone; KRd, carfilzomib, lenalidomide, dexamethasone; KCd, carfilzomib, cyclophosphamide, dexamethasone.

*Other arm (KCd) not presented here.

Newer studies which have included the addition of anti-CD38 antibody agents to a triplet backbone have led to deep responses when used together with AHCT and are summarized in **Table 3**. Of note, none of these quad-therapy studies have compared upfront AHCT to a deferred- or non-AHCT control arm. In CASSIOPEA, Dara-DTd plus AHCT induced excellent response rates and MRD negativity compared to DTd plus AHCT alone, with a 93% 2-year PFS in the quad-containing arm (67). The phase II GRIFFIN trial tested Dara-VRd as induction with AHCT and showed similarly excellent 2-year PFS at 95.8% (68). The recently published phase III PERSEUS trial compared Dara-VRd vs VRd in induction and post-AHCT consolidation, and demonstrated a significant improvement in PFS with the addition of Dara (84.3% 4-year PFS, vs 67.7% in the VRd only group) (6). Importantly PERSEUS reported a powerful depth of response with 75.2% of patients in the D-VRd group achieving MRD-negativity. Similarly, the phase III IsKia trial compared the combination of Isatuximab-KRd to KRd alone with AHCT and consolidation showed significant improvement in MRD negativity rates with the quadruplet (MRD at 10^-5^ 77% vs 67%; MRD at 10^-6^ 67% vs 48% respectively) (69). It is important to consider whether the benefits seen from these therapies are attributable more to the addition of the anti-CD38 agent itself, as opposed to the quad-agent nature of combination. Indeed, more drugs are not necessarily better, as demonstrated in trials of other four-drug inductions outside the anti-CD38 setting, suggesting they may perform as well as three-drug combinations (70). Additionally, the MAIA trial showed a significant PFS and OS with the addition of daratumumab to Rd in transplant ineligible patients (71), though this abridged regimen has not been studied in the AHCT setting.

**Table 3:** Benefit of anti-CD38 containing quad-therapy in newly diagnosed myeloma.

	Use of AHCT	Induction regimen	PFS	OS	MRD-Negative Rate (%, time-point, sensitivity)
**CASSIOPEA**	All arms received upfront AHCT	Dara-VTd	93% 2-year PFS	Not reported	64% at 100 days post-AHCT (10^-5^)
**GRIFFIN**	All arms received upfront AHCT	Dara-VRd	95.8% 2-year PFS	92.7% 4-year OS:	Post-induction: 22%/1% Post-consolidation: 50%/11% Post-1-year-maintenance: 59%/21% End of study: 64%/36% (10^−5^/10^−6^)
**PERSEUS**	All arms received upfront AHCT	Dara-VRd	84.3% 4-year PFS	Not reported	75%/65% any timepoint during study (10^-5^/10^-6^) 64.8% sustained negativity for ≥12months (10^-5^)
**MASTER**	All arms received AHCT	Dara-KRd	87% 2-year PFS	94% 2-year OS	81%/71% at post-consolidation (10^-5^/10^-6^)
**MANHATTAN**	No AHCT	Dara-KRd	98% 1-year PFS	100% 1-year OS	71% post-cycle 8 (10^-5^)

Dara-VRd, daratumumab, bortezomib, lenalidomide, dexamethasone; Dara-VTD, daratumumab, bortezomib, thalidomide, dexamethasone; Dara-KRd, daratumumab, carfilzomib, lenalidomide, dexamethasone.

### 3.1 Impact of disease risk

Cytogenetics, and more recently MRD status, have been shown to correlate with survival. In a 2022 evaluation of the impact of AHCT with quad-therapy induction in NDMM, MRD was assessed by NGS pre and post AHCT, and the group with the greatest reduction in MRD burden had high-risk cytogenetics (HRCG) demonstrating a ‘dose effect’ with stepwise greater reduction in those with 0, 1 or 2+ HRCG abnormalities (72). Those with more than 2 HRCG abnormalities – so called ultra-high risk – have worse outcomes as demonstrated in subgroup analysis of MASTER and GRIFFIN trials (73). Though among ultra-high risk patients, those who achieve MRD negativity prior to or after AHCT have improved outcomes (74). In IFM 2009 long term follow up subgroup analysis, PFS (HR 0.28, p<0001) and OS (HR 0.35, p<0.001) was longer in patients who became MRD negative (64), and in DETERMINATION, there was no PFS difference between AHCT and non-AHCT therapy in patients who achieved MRD negativity (59). (59). In the CARDAMON trial, of the 22.8% of patients who achieved MRD negativity following induction, analysis suggested there was no benefit from AHCT gained in this group (66). A large retrospective study of NDMM patients who achieved a VGPR or greater after induction therapy assessed the MRD status by next-generation flow cytometry and found pre-AHCT MRD positivity was associated with a shorter PFS (48.2 months vs 80.1 months, p<0.001) (75). Finally, the single-arm MASTER trial attempted to use MRD negativity to guide decision making in patients receiving Dara-KRd induction followed by upfront AHCT and Dara-KRd consolidation, ceasing treatment when a patient achieved two consecutive MRD-negative readings. This strategy showed promising PFS and rates of MRD negativity (76). HRCG patients in the MASTER trial had far poorer PFS, especially when their therapy was stopped-and achievement of MRD negativity.

### 3.2 Role of autograft at relapse

There have been several retrospective studies evaluating responses to a second or third AHCT in the setting of RRMM, demonstrating this a feasible and safe approach, which may provide PFS benefit (77–82). Further retrospective subgroup analysis studies have demonstrated the benefit of salvage AHCT is greater in those who had a longer duration of response with their first AHCT (83, 84). This was recently called into question when long term follow-up of the GMMG ReLApsE trial did not show a difference in PFS or OS, but patients were not allowed onto the study if lenalidomide refractory, and therefore likely not generalizable to the current RRMM population (85). The interim analysis of the prospective single arm Second Chance trial shows deep responses with median PFS not reached when using Dara-KRD with salvage AHCT in the early relapse setting (86).

Melphalan retains its potent disease control even in the post CAR T setting. In a recent assessment of salvage therapies after relapse following BCMA-directed CAR T cell treatment, there appeared to be a reasonable response with 71.4% ORR, and an OS of 23.2 months in those who underwent AHCT or allogeneic HCT. Many of these patients were refractory to multiple lines of therapy (median 5 lines prior to CAR T) and the vast majority (94.9%) had had a prior AHCT (87). Salvage AHCT holds theoretical appeal in augmenting the biology of relapsed myeloma to wipe the slate clean of a heavily exposed patient. The rationale here is twofold: to gain clonal control and reset the immune milieu (88). There is a pattern of immune dysregulation and microenvironment abnormalities seen in myeloma patients with reduced NK and T cells and increased immunosuppressive cells, particularly T regulatory cells (89). This dysfunction worsens with exposure to anti-myeloma agents (90, 91). With an infusion of relatively chemotherapy naïve autologous stem cells, there opens up an opportunity for myeloma-specific immunity to be regained. In particular, the pattern of dynamics of T cell reconstitution after AHCT with a favorable ratio of T regulatory to T effector cells (92–94), may be able to be harnessed to leverage the sensitivity to immune therapies including CAR T (95). This is being tested prospectively prior to CAR T cells in NCT05393804 with the hypothesis that “fresh” non-exhausted T cells will lead to better expansion and persistence of the CAR T cell made from these cells. Furthermore, the early recovery of NK cells after AHCT may provide an opportunity to maximize potency of NK cell-therapies in this window (96).

## 4 Discussion: CAR T or AHCT or both?

It remains very difficult to show OS benefit in any modern comparative trial for MM given the median 7-10 year survival quoted for standard risk patients and significant crossover that occurs in many trials. Increasingly, patients’ OS is based on sequential progression free intervals in which the optimal sequence is unclear and ever changing due to newer data, approvals, and guidelines (97–99). The considerations we present in this section presume the indications approved in the United States in early 2024, and we acknowledge that in other parts of the world, these discussions differ based on availability and cost (100–102).

Firstly, studies of delayed AHCT, performed >12 months after diagnosis, suggested a reasonable response to this approach with a similar median time to progression and no difference in OS rates (5, 59). A major issue seen in the IFM2009 study was that 21% of those randomized to delayed AHCT -and deemed transplant eligible at randomization - were not able to later receive a salvage AHCT (5). A more recent retrospective comparison of upfront or delayed AHCT, found that delayed AHCT did not result in worse OS or PFS even when adjusting for age, disease risk, or depth of response at time of collection, but interestingly highlighted that those who underwent delayed AHCT frequently received a lower melphalan dose, reflective of mounting medical complexity with the passage of time and disease evolution (103). Data on the outcomes and safety of CAR T in frail patients suggests a relatively tolerable profile in this group, giving some weight to the argument that reserving CAR T for later in a patient’s course may be a more deliverable sequence (104, 105).

Second, some believe that the post CAR T cell journey is much easier than after AHCT, but this may not always be the case. Prolonged cytopenias, immune compromise, CRS, ICANS, MNTs, and infection risk, and the requirements to stay within a certain distance of the treating facility can impact qualify of life (QoL) after CAR T infusion. Comparisons show that the recovery to baseline may not be that different between the two modalities (106, 107).

Increasingly concerning is the risk of secondary malignancies. A CIBMTR analysis recently reported a risk of 4% at a median of 37 months of follow-up after AHCT, and though most of these patients eventually died from their myeloma rather than the secondary malignancy, these patients had a reduced PFS and OS (108). However, studies have also demonstrated that melphalan exposure and AHCT (+/- lenalidomide exposure) increase the mutational burden in patients with MM (109, 110). On the CAR T side, the updated analysis of CARTITUDE-1 showed 16/97 (16%) had a secondary malignancy with 9 (9%) being myelodysplastic syndrome or acute myeloid leukemia (42). It true risk of CAR T derived T cell lymphoma is not yet clear, and impacts on monitoring guidelines yet to be established (55, 111). The etiology of these findings, and whether it may manifest with earlier use of CAR T are not yet known.

Practical and financial considerations will inevitably shape the uptake of these therapies, and incremental cost effectiveness analysis should be factored into paradigm development. CAR T therapy costs are known to be dependent on rates of CRS and ICANS, and resource requirements may be prohibitive in some settings (112, 113). AHCT and CAR T costs may be reduced with utilization of outpatient care packages, however institutions need to have the resources and quality systems in place in order to safely facilitate the delivery of outpatient care, which can be a limiting factor particularly in low- and middle-income countries (LMIC) (114). In the LMIC setting, uptake of more efficacious practice may be limited at least in the short-term by costs, and we should be mindful of the increasing gap of resource-intensive and high-cost practices between high-, middle- and low-income settings (115, 116). Short-term focus can be premature however, and recent analyses have suggested more intensive therapies upfront may not only offset costs but leads to a long-term cost savings (117). Given the chronic nature of MM, our continued improvement in managing side effects, shortening hospital length of stay, and generally improving safety of both AHSCT and CAR T will be increasingly important to consider when evaluating the economic and quality of life impact. This will be especially important as CAR T migrates into less academic institutions where the systems to ensure adequate supportive care may need to be optimized. Additionally, when considering the prospect of bringing CAR T therapy to earlier lines of treatment, we will need to understand the value beyond traditional efficacy alone, with demonstration of quality-adjusted life years and other patient-reported outcomes, and the cost (both short- and long-term) to the healthcare system (97).

Overall, patients with MM will likely have both CAR T and AHCT during their treatment course. Sequencing depends on approvals and availability of the options, and will change over time as more treatments are available in earlier lines and with the results for the frontline prospective studies mentioned above. Prior toxicities and comorbidities, as well as concerns for future determents to quality of life and risk of secondary malignancies, allow for discussion and personalization of treatment. Optimizing both of these very effective modalities can allow patients to have long progression free remissions, which may even allow for a yet undescribed curative mechanism of action therapy to be approved.

## Author contributions

CH: Writing – original draft, Writing – review & editing. GS: Conceptualization, Supervision, Writing – review & editing. BP: Conceptualization, Supervision, Writing – review & editing, Writing – original draft.
